# N_2_-fixation and N contribution by grain legumes under different soil fertility status and cropping systems in the Guinea savanna of northern Ghana

**DOI:** 10.1016/j.agee.2017.08.028

**Published:** 2018-07-01

**Authors:** M. Kermah, A.C. Franke, S. Adjei-Nsiah, B.D.K. Ahiabor, R.C. Abaidoo, K.E. Giller

**Affiliations:** a Plant Production Systems, Wageningen University, P.O. Box 430, 6700 AK Wageningen, The Netherlands; b Soil, Crop and Climate Sciences, University of the Free State, P.O. Box 339, Bloemfontein 9300, South Africa; c International Institute of Tropical Agriculture, P.O. Box TL 06, Tamale, Ghana; d CSIR-Savanna Agricultural Research Institute, P.O. Box 52, Tamale, Ghana; e Department of Theoretical and Applied Biology, Kwame Nkrumah University of Science and Technology, Kumasi, Ghana

**Keywords:** Cowpea, Soybean, Groundnut, Maize, Partial N balance

## Abstract

Continuous cereal-based cropping has led to a rapid decline in soil fertility in the Guinea savanna agro-ecological zone of northern Ghana with corresponding low crop yields. We evaluated the effects of cropping system and soil fertility status on grain yields and N_2_-fixation by grain legumes and net N contribution to soil fertility improvement in contrasting sites in this agro-ecological zone. Maize was intercropped with cowpea, soybean and groundnut within a row, with a maize stand alternated with two equally spaced cowpea or groundnut stands and in the maize-soybean system, four equally spaced soybean stands. These intercrops were compared with sole crops of maize, cowpea, soybean and groundnut in fertile and poorly fertile fields at sites in the southern (SGS) and the northern (NGS) Guinea savanna. The proportion of N derived from N_2_-fixation (%Ndfa) was comparable between intercrops and sole crops. However, the amount of N_2_-fixed was significantly larger in sole crops due to a greater biomass accumulation. Legumes in poorly fertile fields had significantly smaller shoot *δ*^15^N enrichment (−2.8 to +0.7‰) and a larger %Ndfa (55–94%) than those in fertile fields (−0.8 to +2.2‰; 23–85%). The N_2_-fixed however was larger in fertile fields (16–145 kg N ha^−1^) than in poorly fertile fields (15–123 kg N ha^−1^) due to greater shoot dry matter and N yields. The legumes grown in the NGS obtained more of their N requirements from atmospheric N_2_-fixation (73–88%) than legumes grown in the SGS (41–69%). The partial soil N balance (in kg ha^−1^) was comparable between intercrops (−14 to 21) and sole legumes (−8 to 23) but smaller than that of sole maize receiving N fertiliser (+7 to +34). With other N inputs (aerial deposition) and outputs (leaching and gaseous losses) unaccounted for, there is uncertainty surrounding the actual amount of soil N balances of the cropping systems, indicating that partial N balances are not reliable indicators of the sustainability of cropping systems. Nevertheless, the systems with legumes seem more attractive due to several non-N benefits. Our results suggest that soybean could be targeted in the SGS and cowpea in the NGS for greater productivity while groundnut is suited to both environments. Grain legumes grown in poorly fertile fields contributed more net N to the soil but growing legumes in fertile fields seems more lucrative due to greater grain and stover yields and non-N benefits.

## Highlights

• Cropping system and soil fertility effects on N_2_-fixation were tested in northern Ghana.
• More N_2_ is fixed in sole cropping than intercropping despite comparable %Ndfa.
• Poorly fertile fields give limited grain legume benefits despite enhanced %Ndfa.
• Partial N balances are unreliable indicators of cropping system sustainability.
• Different grain legumes should be targeted to different sites in the Guinea savanna.

## 1 Introduction

The Guinea savanna agro-ecological zone of northern Ghana is characterised by a single cropping season (with 180–200 growing days), a unimodal rainfall pattern and an annual mean precipitation of 1100 mm (SRID, 2016). The soils in many parts of the region are poor in fertility, particularly N (Dakora et al., 1987). Shortened fallow periods have exerted pressure on the already fragile soils (Dakora et al., 1987, Franke et al., 2004). These issues, compounded by continuous cereal-based systems without sufficient nutrient inputs to the soil, have led to wide scale declines in soil fertility and persistently poor crop yields on smallholder farms (Sanginga, 2003).

The incorporation of grain legumes into cereal-based cropping systems can contribute to the replenishment of soil fertility through the fixation of atmospheric nitrogen (N_2_), while supplying protein-rich grains for household food and nutrition (Giller, 2001). In the West African Guinea savanna, grain legumes fix between 15 and 201 kg N ha^−1^ per season (Dakora et al., 1987, Sanginga et al., 1997, Belane and Dakora, 2010, Yusuf et al., 2014). A net N contribution of up to 48 kg ha^−1^ by groundnut (Yusuf et al., 2014) and 125 kg N ha^−1^ by cowpea (Dakora et al., 1987) with the grain exported from the field has been documented. Consequently, incorporation of grain legumes into cereal-based cropping systems represents an opportunity to address these soil fertility concerns. Legumes can be incorporated through sole-cropped legume-cereal rotations as predominantly practised by farmers in the region. However, the increased risk of crop failure in sole cropping due to an unpredictable rainfall regime in the single cropping season threatens household food security. Accordingly, intercropping the main cereals (especially maize which is the dominant crop in the area) with grain legumes can alleviate such risks to safeguard household food and income security (Giller, 2001).

The high labour requirements and the general yield reduction of the main crop in cereal-legume intercropping compared with sole cropping are a concern for farmers. Nevertheless, cereal-legume intercropping may improve diversification in nutrient uptake by the component crops, environmental resources use efficiencies and increased yield per unit area relative to sole cropping (Willey, 1990). Cereal-legume intercropping thus presents an alternative to sole cropping. The diverse bio-physical environments and variable crop management strategies lead to a large variability in benefits from N_2_-fixation and net N contribution of legumes to the soil (Giller, 2001). Also, grain legume species and varieties differ in their contribution to soil N fertility enhancement (Giller, 2001). This suggests a need for targeting different legume species to different agro-ecological zones or contrasting environments within an agro-ecological zone for increased yields and soil fertility improvement.

Several studies have quantified N_2_-fixation and net N contribution to the soil in the Guinea savanna of West Africa (e.g. Eaglesham et al., 1981, Sanginga et al., 1997, Ogoke et al., 2003, Yusuf et al., 2008) and northern Ghana (e.g.Dakora et al., 1987, Naab et al., 2009, Belane and Dakora, 2010, Konlan et al., 2015). Only few studies (e.g. Eaglesham et al., 1981, Konlan et al., 2015) assessed the effect of maize-grain legume intercropping on N_2_-fixation. Even so, the net N contributions to the soil from the intercrop systems were not measured. In addition, the wide variability in soil fertility across the different fields in the West African Guinea savanna agro-ecological zone was not considered. The objectives of this study were to determine the effects of: (i) intercropping, (ii) soil fertility status and (iii) grain legume species on grain yield, N_2_-fixation and net N contribution to soil fertility improvement in the southern and northern Guinea savanna agro-ecological zones of northern Ghana.

## 2 Materials and methods

### 2.1 On-farm trials and trial management

The field trials were conducted on-farm in the 2013 cropping season at Kpataribogu {9°58′ N, 0°40′ W; 172 m above sea level (masl)} in the Karaga District (southern Guinea savanna, SGS) and at Bundunia (10°51′ N, 1°04′ W; 185 masl) in the Kassena-Nankana East Municipal (northern Guinea savanna, NGS) of northern Ghana. Rainfall was recorded with rain gauges at both trial sites. A total of 598 mm in the SGS and 532 mm rainfall in the NGS were received during the growing season. The soils at both sites are classified as Savanna Ochrosol and Groundwater Laterites in the interim Ghana soil classification system (Adjei-Gyapong and Asiamah, 2002) and as Plinthosols in the World Reference Base for soil resources (WRB, 2015). Two field types representing fertile and poorly fertile soil conditions were selected at each site, using farmers’ knowledge and the help of agricultural extension officers. Fields selected were under mono-cropped maize, grain legume or cotton in the three preceding seasons. Soils of each field were sampled at 0–15 cm depth prior to land preparation, thoroughly mixed and about 1 kg sub-sample was air-dried, sieved through a 2 mm-mesh sieve and analysed for pH (1:2.5 soil:water suspension), organic C (Walkley and Black), total N (Kjeldahl), available P (Olsen), exchangeable K, Mg, and Ca (in 1 M ammonium acetate extracts) and texture (hydrometer method).

**Fig. 1:**
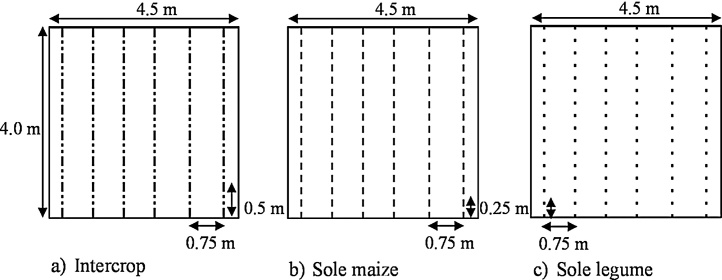
Schematic overview of cropping patterns: a) maize-legume within-row intercrop treatment, b) sole maize treatment and c) sole legume treatment. The intercrop scheme shown is for maize-cowpea and maize-groundnut systems. For the maize-soybean intercrop, a maize stand was alternated with four soybean stands within a row. Sole legume scheme (Fig. 1c) is for sole crops of cowpea and groundnut (16 plant stands per row). Sole soybean treatment had 32 plant stands per row (0.125 m intra-row spacing).

Treatments consisted of cowpea – *Vigna unguiculata* (L.) Walp; soybean *– Glycine max* (L.) Merr. and groundnut – *Arachis hypogaea* L. intercropped with maize (*Zea mays* L.) or grown as sole crops. In the intercrop treatments, maize and legumes were grown within the same row. A maize stand was alternated with two equally spaced cowpea or groundnut stands within a row. In the maize-soybean system, a maize stand was alternated with four soybean stands within a row. Maize and all intercropped legumes were sown at one seed per hill, while sole legumes were sown at two seeds per hill. Inter-row spacing was 75 cm in all treatments. Intercropped maize was spaced at 50 cm within a row while intra-row spacing for sole maize was 25 cm. Sole cowpea and groundnut had an intra-row spacing of 25 cm and that of sole soybean was 12.5 cm. These resulted in plant populations (plants ha^−1^) of 26,667 and 53,333 for maize, 53,333 and 106,666 for cowpea and groundnut, and 106,666 and 213,334 for soybean, respectively for intercrops and sole crops. The spatial planting arrangements of the different cropping patterns are shown in Fig. 1. The experimental design was a randomised complete block design. Blocks of treatments were replicated four times per fertility level at each site and treatments were randomised within blocks. A single plot measured 4.5 × 4.0 m.

The land was tractor-ploughed, ridged and sowing done on the apex of the ridges. The varieties used were Padi-tuya: SARC 3-122-2 (cowpea), Jenguma: Tgx 1448-2E (soybean), Samnut 22 (groundnut) in SGS and Chinese variety (groundnut) in NGS, and Obatanpa: GH83-63SR (maize). All crops were sown on July 1–2 in the SGS and July 16–17 in the NGS. Sowing was relatively late due to a late onset of rains. Soybean seeds were inoculated with the commercial inoculant Legumefix (Legume Technology, UK) containing *Bradyrhizobium japonicum* strain 532c (re-isolated in Brazil from strain USDA 442 Wisconsin, USA) at sowing at the rate of 5 g of inoculant per kg of seed. At sowing, 25 kg P ha^−1^ and 30 kg K ha^−1^ as TSP and MoP were uniformly applied to all treatments. Urea was spot-applied to only maize stands at a rate of 25 kg N ha^−1^ for intercropped maize and 50 kg N ha^−1^ for sole maize. Half of the N was applied at three weeks after sowing (WAS) and the other half at six WAS. All fertilisers were band-applied at 3 cm depth and 5 cm from the plants. No N fertiliser was applied to sole legumes. Plots were weeded twice with hoe at 3 and 6 WAS.

### 2.2 Yields, N_2_-fixation and N uptake measurements

Legume shoot biomass was sampled at mid-pod filling stage from a 3.0 m^2^ subplot, separated into shoots and pods and both the total and sub-sample fresh weights were taken in the field. Grain and stover yields were assessed from a 4.5 m^2^ subplot at crop maturity with both total and sub-sample fresh weights taken in the field. Fresh to dry weight conversion factors were used to convert the sub-sample fresh weights to dry weights: Cowpea (biomass harvest at mid-pod-filling: shoot = 0.17, pod = 0.18; harvest at crop maturity: haulm = 0.19, pod = 0.64, grain to pod ratio = 0.77, husk to pod ratio = 0.23), soybean (biomass harvest at mid-pod-filling: shoot = 0.29, pod = 0.31; harvest at crop maturity: haulm = 0.91, pod = 0.69, grain to pod ratio = 0.71, husk to pod ratio = 0.29), groundnut (biomass harvest at mid-pod-filling: shoot = 0.22, pod = 0.31; harvest at crop maturity: haulm = 0.34, pod = 0.66, grain to pod ratio = 0.64, husk to pod ratio = 0.36) and maize (harvest at crop maturity: haulm = 0.38, cob = 0.71, grain to cob ratio = 0.79, core to cob ratio = 0.21). These were derived from experimental data by taking pooled means of several treatments and have previously been reported by Kermah et al. (2017). Legume and maize grain yields are presented at 12% and 14% moisture content, respectively, shoot biomass and stover yields on a dry weight basis. Stover yield includes both the haulms and the husks.

Non-legume broad-leaved weeds growing along the borders of the main plots were sampled from each block and used as reference plants for estimating N_2_-fixation using the ^15^N natural abundance method (Unkovich et al., 2008). Several reference weed species were collected per block and the mean *δ*^15^N enrichment of these reference species was used in estimating the proportion of N derived from atmosphere (%Ndfa). The weighted *δ*^15^N of whole shoots was calculated from the separate *δ*^15^N measurements of shoots and pods harvested at mid-pod filling and used to estimate %Ndfa.

As N concentrations in legume grain and stover at maturity were not measured, legume N uptake was estimated with mean N concentrations taken from Nijhof (1987): cowpea grain: 2.90%, cowpea stover: 1.73%; soybean grain: 6.10%, soybean stover: 1.05%; groundnut grain: 4.50%, groundnut stover: 1.40%. For maize, N concentrations in grain and stover measured from experimental plots in an adjacent trial at each site (with the same maize variety and similar fertiliser treatment as in our trial) were used to calculate N uptake: in the SGS maize grain: 1.46%, maize stover: 0.63% and in the NGS, maize grain: 1.41%, maize stover: 0.55. The C:N ratios were calculated assuming that the carbon concentration in the crop residues was 40% (Partey et al., 2014).

### 2.3 Calculations and statistical analysis

The weighted *δ*^15^N for whole shoot was calculated as:

(1) {(shoot N × *δ*^15^N shoot) + (pod N × *δ*^15^N pod)}/(shoot N + pod N)

Shoot N = %N shoot/100 × shoot dry matter yield (kg ha^−1^); Pod N = %N pod/100 × pod dry matter yield (kg ha^−1^). %N derived from N_2_-fixation (%Ndfa) was calculated from the weighted *δ*^15^N values using the equation of Unkovich et al. (2008) as:

(2) %Ndfa = {(*δ*^15^N _ref_ − *δ*^15^N _leg_)/(*δ*^15^N _ref_ − B)}100

where *δ*^15^N_ref_ and *δ*^15^N _leg_ are the *δ*^15^N natural abundance of the shoots of the non-N_2_-fixing reference plants (fully dependent on N from the soil) and the *δ*^15^N natural abundance of the N_2_-fixing legumes, respectively; and B is the *δ*^15^N of shoots of the test legume fully dependent on N_2_-fixation (a measure of isotopic fractionation during N_2_-fixation). The smallest weighted *δ*^15^N value for each legume shoot was used as the B value (Peoples et al., 2002): i.e. cowpea: −3.52; soybean: −2.04 and groundnut: −0.71. Shoot N_2_-fixed (kg ha^−1^) was calculated as:

(3) Shoot N_2_-fixed (kg ha^−1^) = %Ndfa × whole shoot N

The amount of N_2_-fixed in the whole plant as reported in this paper was calculated assuming that 30% of N_2_ fixed was present in the roots (Unkovich et al., 2008):

(4) Total N_2_-fixed (kg ha^−1^) = shoot N_2_-fixed/(0.70)

Eq. (4) was used to estimate the total amount of N_2_-fixed for soil N balance determination as the inclusion of the N_2_-fixed in below-ground dry matter has a significant impact on the soil N balance (Peoples et al., 2009).

The net N (kg ha^−1^) contribution to the soil N economy was calculated in two scenarios as:

(5) Scenario 1 (only grain exported):

(i) Intercrop = total N_2_-fixed + applied N − legume grain N − maize grain N

(ii) Sole legume = total N_2_-fixed − grain N

(iii) Sole maize = applied N − grain N

(6) Scenario 2 (grain + stover exported):

(i) Intercrop = total N_2_-fixed + applied N − legume grain N − legume stover N − maize grain N − maize stover N

(ii) Sole legume = total N_2_-fixed − grain N − stover N

(iii) Sole maize = applied N − grain N − stover N

Statistical analysis was conducted using GenStat (version 18.1, VSN International Ltd). Data were analysed with a linear mixed model. For each legume species, data for both cropping systems and soil fertility status were analysed together for each site with cropping system and soil fertility as fixed factors and replication as a random factor. To test for the effect of legume species on shoot *δ*^15^N and %Ndfa, data for both cropping systems across fertility status for all three legume species were analysed together per site with cropping system, fertility and legume species as fixed factors and replication as random factor. For cross-site analysis, data for all cropping systems across fertility status for each legume species for both sites were analysed together with all factors including site kept fixed and replication as random factor. Both individual and interaction effects of these factors on N_2_-fixation and soil N balance were analysed. The standard error of differences between means (SED) was used to compare treatment means at a significance level of *P* < 0.05.

## 3 Results

### 3.1 Soil fertility classification

The soil analysis confirmed the farmers’ soil fertility classification at both sites (Table 1). The fertile fields had superior soil fertility parameters than the poorly fertile fields at both sites. In the SGS, the fertile field had favourable OC, P, exchangeable Ca and ECEC while in the NGS, pH, OC, N, P, Ca and ECEC were more favourable for crop growth in the fertile field. At both sites however, available P was low and likely to limit crop growth without the application of P fertiliser. The soils in the SGS had better fertility characteristics, particularly a higher OC, N and ECEC, than the soils in the NGS which were more sandy and acidic. Soil available P and exchangeable cations were similar at both sites.

**Table 1:** Physico-chemical properties of the experimental fields differing in soil fertility in the southern Guinea savanna (SGS) and northern Guinea savanna (NGS) of northern Ghana.

Soil fertility parameter	SGS		NGS	
	Fertile field	Poorly fertile field	Fertile field	Poorly fertile field
pH	6.2	5.8	5.4	4.7
Organic C (g kg^−1^)	10.9	7.4	6.2	3.9
Total N (g kg^−1^)	0.9	0.8	0.6	0.2
Olsen P (mg kg^−1^)	2.6	1.7	2.8	1.9
K (cmol_+_ kg^−1^)	0.3	0.2	0.2	0.1
Ca (cmol_+_ kg^−1^)	1.7	1.3	1.6	0.8
Mg (cmol_+_ kg^−1^)	0.7	0.7	0.9	0.7
ECEC (cmol_+_ kg^−1^)	10.2	5.2	6.9	3.0
Sand (g kg^−1^)	563	538	738	798
Silt (g kg^−1^)	321	400	160	160
Clay (g kg^−1^)	116	61	101	41

### 3.2 δ^15^N enrichment of reference weed species

In the NGS, significant differences (*P* = 0.019) were observed in the *δ*^15^N enrichment of the different weed reference species used to estimate the %Ndfa (Table 2). The *δ*^15^N values differed between soil fertility status (*P* < 0.001 in SGS; *P* = 0.029 in NGS), with larger values in the fertile fields. Averaged over species and soil fertility levels, *δ*^15^N values were significantly larger in the SGS than in the NGS (Table 2).

**Table 2:** The *δ*^15^N natural abundance (‰) in different species of broad-leaved non-N_2_-fixing reference plants and grain legumes (as affected by cropping system) at different soil fertility status at sites in southern Guinea savanna (SGS) and northern Guinea savanna (NGS) of northern Ghana.

Plant species	SGS		NGS	
	*δ*^15^N (‰)	Range *δ*^15^N (‰)	*δ*^15^N (‰)	Range*δ*^15^N (‰)
*Fertile field*				
Non-N_2_-fixing reference weeds				
*Hyptis spicigera*			4.0	1.7–6.1
*Borreria scabra*	5.9	5.9	1.5	1.5
*Mitracarpus villosus*	4.0	2.7–6.8		
*Aspilia bussei*	5.9	4.5–6.8	2.9	0.9–7.6
*Commelina benghalensis*	3.9	3.0–4.7		
*Acanthospermum hispidium*			4.0	2.5–6.0
*Leucas martinicensis*			3.9	3.2–4.6
Legumes				
Intercrop CP	2.2	1.2–3.5	−0.8	−1.1 to −0.5
Sole CP	1.8	0.8–3.6	−0.8	−1.4 to −0.2
Intercrop SB	−0.5	−0.9 to 0.3	−0.7	−1.1 to −0.2
Sole SB	−0.3	−0.9 to 0.5	−0.3	−0.6 to 0.03
Intercrop GN	0.9	0.6–1.7	0.1	−0.3 to 0.8
Sole GN	1.4	1.0–1.9	−0.1	−0.1 to 0.04

*Poorly fertile field*				
Non-N_2_-fixing reference weeds				
*Hyptis spicigera*	2.9	2.2–3.6	3.8	2.5–5.5
*Borreria scabra*	3.3	1.1–5.1	1.8	0.9–3.0
*Mitracarpus villosus*	1.9	1.4–2.4	2.2	1.0–3.9
*Aspilia bussei*	3.3	1.9–5.8		
*Commelina benghalensis*	4.3	3.7–4.7		
Legumes				
Intercrop CP	0.1	−0.3 to 0.6	−2.6	−2.7 to −2.5
Sole CP	0.1	−1.3 to 1.2	−2.8	−3.5 to −2.4
Intercrop SB	−0.5	−1.4 to 0.8	−1.0	−1.6 to −0.2
Sole SB	−0.5	−1.2 to 0.1	−1.7	−2.0 to −1.4
Intercrop GN	0.1	−0.1 to 0.3	−0.5	−0.7 to −0.4
Sole GN	0.7	0.1–1.8	−0.4	−0.5 to −0.3
*SED (weed species)*	*n.s.*		0.89*	
*SED (legume species)*	0.27***		0.12***	
*SED (fertility effect weeds)*	0.41**		0.48*	
*SED (fertility effect legumes)*	0.25**		0.13***	
*SED (cropping system)*	*n.s.*		*n.s.*	
*SED (all plant species)*	0.40***		0.41***	

SED = combined standard error of differences between means for: weed species across fertility; legume species across fertility; fertility across weed species or legume species; cropping system across fertility; and all plant species (both legumes and weed species combined).

* Significant at *P* < 0.05.

** Significant at *P* < 0.01.

*** Significant at *P* < 0.001.

### 3.3 Shoot biomass, grain and stover yields

Legume shoot dry matter and shoot N yields were in most cases significantly larger in sole crops than intercrops (Table 3). Legumes in fertile fields provided significantly greater shoot dry matter and N yields of cowpea at both sites, while that of soybean was superior in the fertile field in the NGS only (Table 3). Mean soybean shoot dry matter and N yields were 1066 kg ha^−1^ and 28 kg N ha^−1^ significantly greater in SGS, while shoot dry matter of cowpea was 349 kg ha^−1^ significantly larger in NGS but shoot N yield was rather 9 kg ha^−1^ less in the NGS. Groundnut shoot dry matter (<1 t ha^−1^) and N yield (max 31 kg ha^−1^) were low at both sites.

**Table 3:** The proportion of N derived from N_2_-fixation (%Ndfa), shoot dry matter, whole shoot N and total N_2_-fixed by cowpea (CP), soybean (SB) and groundnut (GN) measured at mid-pod filling stage at different soil fertility status and cropping systems in the southern Guinea savanna (SGS) and northern Guinea savanna (NGS) of northern Ghana.

Fertility status	Cropping system	SGS				NGS			
		Ndfa (%)	Shoot dry matter (kg ha^−1^)	Shoot N (kg ha^−1^)	N_2_-fixed (kg ha^−1^)	Ndfa (%)	Shoot dry matter (kg ha^−1^)	Shoot N (kg ha^−1^)	N_2_-fixed (kg ha^−1^)
Fertile	Intercrop CP	23	1741	56	16	64	2147	47	43
	Sole CP	29	2449	75	31	64	2806	60	54
Poorly	Intercrop CP	55	743	23	18	83	878	15	17
fertile	Sole CP	56	640	21	17	87	1135	19	23
	*SED*^a^*(system)*	*n.s.*	61**	3*	*n.s.*	*n.s.*	196*	*n.s.*	*n.s.*
	*SED*^b^*(fertility)*	11*	161***	5***	*n.s.*	4**	191***	3***	4***

Fertile	Intercrop SB	77	3344	89	97	67	3563	96	92
	Sole SB	74	4909	136	145	57	5413	156	127
Poorly	Intercrop SB	63	2643	72	67	75	1272	30	33
fertile	Sole SB	62	5448	142	123	92	1833	45	57
	*SED*^a^*(system)*	*n.s.*	469***	12**	14**	*n.s.*	380*	13*	13*
	*SED*^b^*(fertility)*	*n.s.*	*n.s.*	*n.s.*	*n.s.*	4**	559**	15***	15**

Fertile	Intercrop GN	58	776	23	19	81	579	17	19
	Sole GN	48	963	28	21	85	953	31	38
Poorly	Intercrop GN	73	518	14	15	94	570	16	22
fertile	Sole GN	54	957	28	21	92	839	25	33
	*SED*^a^*(system)*	*n.s.*	*n.s.*	*n.s.*	*n.s.*	*n.s.*	110*	3**	4**
	*SED*^b^*(fertility)*	*n.s.*	*n.s.*	*n.s.*	*n.s.*	4*	*n.s.*	*n.s.*	*n.s*

*Prob. F. for site comparisons (cowpea)*: %Ndfa (*P* < 0.001), Shoot dry matter (*P* = 0.016), Shoot N (*P* = 0.0014), N_2_-fixed (*P* = 0.021).

*Prob. F. for site comparisons (soybean)*: %Ndfa (n.s.), Shoot dry matter (*P* = 0.013), Shoot N (*P* = 0.009), N_2_-fixed (*P* = 0.010).

*Prob. F. for site comparisons (groundnut)*: %Ndfa (*P* < 0.001), Shoot dry matter (n.s.), Shoot N (n.s.), N_2_-fixed (*P* = 0.010).

a Combined SED for cropping system across soil fertility.

b Combined SED for soil fertility.

* Significant at *P* < 0.05.

** Significant at *P* < 0.01.

*** Significant at *P* < 0.001.

Intercropping significantly reduced grain yields of all three legume species and of maize compared with the sole crops in the SGS, but these differences were often not significant in the NGS (Table 4). The influence of soil fertility on grain yield differed among legume species. Only grain yields of cowpea and soybean were larger (*P <* 0.001 generally) in the fertile fields at both sites (Table 4). Maize grain yields were in most cases greater (*P <* 0.01 generally) in the fertile fields at both sites, with a mean of 547 kg ha^−1^ and 806 kg ha^−1^ more maize grain produced in the fertile fields than the poorly fertile fields in the SGS and NGS, respectively (Table 4). Mean cowpea grain yield was 190 kg ha^−1^ significantly greater in the NGS, compared with the yield in the SGS, while soybean and maize grain yields were 267 and 1417 kg ha^−1^, respectively greater in the SGS. Stover yields of cowpea, soybean and maize followed similar trends as grain yields (Table 4). Consistently greater stover yields were obtained in sole cropping and in fertile fields at both sites. Soybean and maize stover yields were significantly greater in the SGS, while that of cowpea was similar between sites. Groundnut grain and stover yields were generally poor at both sites with no difference in grain yield but significantly larger stover yield in the SGS (Table 4).

**Table 4:** Grain, stover yields and harvest index (HI) of cowpea (CP), soybean (SB), groundnut (GN) and maize (MZ) under different soil fertility status and cropping systems at sites in the southern Guinea savanna (SGS) and northern Guinea savanna (NGS) of northern Ghana.

Fertility	Cropping	SGS					NGS				
status	system	Legume grain yield	Legume stover yield	Maize grain yield	Maize stover yield	HI	Legume grain yield	Legume stover yield	Maize grain yield	Maize stover yield	HI
		(kg ha^−1^)	(kg ha^−1^)	(kg ha^−1^)	(kg ha^−1^)	(%)	(kg ha^−1^)	(kg ha^−1^)	(kg ha^−1^)	(kg ha^−1^)	(%)
Fertile	Intercrop CP	1080	1938			36	1570	2465			39
	Sole CP	1456	2519			37	1896	3035			38
	Intercrop MZ			2413	2971	45			1144	1517	43
	Sole MZ			3237	3764	46			1516	1953	44
Poorly	Intercrop CP	596	1200			33	532	1345			28
fertile	Sole CP	683	1646			29	578	1228			32
	Intercrop MZ			1824	2577	41			632	970	39
	Sole MZ			2380	3138	43			711	1253	36
	*SED*^a^*(system)*	95*	124**	100***	171**		72*.	87*	n.s.	n.s.	
	*SED*^b^*(fertility)*	83***	114***	148**	118**		133***	241***	183**	184**	

Fertile	Intercrop SB	1329	1913			41	1666	1669			50
	Sole SB	2206	2994			42	2189	2210			50
	Intercrop MZ			2250	2833	44			1599	1945	45
	Sole MZ			3352	3981	46			2026	2434	45
Poorly	Intercrop SB	849	1084			44	577	662			47
fertile	Sole SB	1882	2796			40	767	922			45
	Intercrop MZ			2116	2685	44			746	1001	43
	Sole MZ			2551	2849	47			787	1266	38
	*SED*^a^*(system)*	132***	192***	162**	222*		n.s.	56***	n.s.	160*	
	*SED*^b^*(fertility)*	104**	n.s	n.s.	243*		150***	137***	68***	66***	

Fertile	Intercrop GN	198	698			22	266	602			31
	Sole GN	359	1371			21	310	664			32
	Intercrop MZ			2532	3220	44			1185	1542	43
	Sole MZ			3056	3572	46			1652	2020	45
Poorly	Intercrop GN	175	686			20	242	496			33
fertile	Sole GN	353	1153			23	237	649			27
	Intercrop MZ			2262	2879	44			696	1229	36
	Sole MZ			2428	3162	43			712	1418	33
	*SED*^a^*(system)*	67*	151*	n.s.	n.s.		n.s.	39*	79*	133*	
	*SED*^b^*(fertility)*	n.s.	n.s.	n.s.	n.s.		n.s.	n.s.	124**	176*	

*Prob. F. for site comparisons (MZ-CP)*: Cowpea grain yield (*P* = 0.032), Cowpea stover yield (n.s.), Maize grain yield (*P* < 0.001), Maize stover yield (*P* < 0.001).

*Prob. F. for site comparisons (MZ-SB)*: Soybean grain yield (*P* = 0.013), Soybean stover yield (*P* < 0.001), Maize grain yield (*P* < 0.001), Maize stover yield (*P* < 0.001).

*Prob. F. for site comparisons (MZ-GN)*: Groundnut grain yield (n.s.), Groundnut stover yield (*P < *0.001), Maize grain yield (*P* < 0.001), Maize stover yield (*P* < 0.001).

a Combined SED for cropping system across soil fertility.

b Combined SED for soil fertility.

* Significant at *P* < 0.05.

** Significant at *P <* 0.01.

*** Significant at *P* <** 0.001.

### 3.4 *δ*^15^N of legumes, %Ndfa and N_2_-fixed

Shoot *δ*^15^N of legumes did not significantly differ between intercrops and sole crops (Table 2). An exception was groundnut in the SGS where the intercrop had a significantly smaller *δ*^15^N. The shoot *δ*^15^N values of legumes were significantly (*P <* 0.001) smaller than that of the non N_2_-fixing reference weeds despite the observed variability in *δ*^15^N enrichment of the reference weeds (Table 2). The *δ*^15^N signatures differed (*P <* 0.001) among legume species. For example, in the SGS, shoot *δ*^15^N was significantly smaller in soybean than in groundnut and cowpea. Legumes on poorly fertile fields had smaller (*P <* 0.01) shoot *δ*^15^N enrichment. Legumes in the NGS with relatively poorer soils (Table 1) had smaller shoot *δ*^15^N enrichment (*P <* 0.001) than in the SGS (Table 2). %Ndfa was not influenced by cropping system but differed (*P <* 0.001) between legume species and sites (Table 3). In the SGS for instance, mean %Ndfa of soybean (69%) and groundnut (58%) was larger (*P <* 0.001) than that of cowpea (41%). In the NGS, %Ndfa was significantly larger in groundnut (88%) than in cowpea (74%) and soybean (73%). %Ndfa was larger (*P <* 0.05) in the poorly fertile fields and in the NGS with relatively poorly fertile fields than in the SGS (Table 3).

The amount of N_2_-fixed by legumes followed a similar trend to shoot dry matter and N yields (Table 3). Sole crops fixed significantly more N_2_ than intercrops. Exceptions were N_2_-fixed by cowpea and groundnut in the SGS which were similar in intercrops and sole crops. The differences in N_2_-fixed between the fertility levels were only significant for cowpea (*P <* 0.001) and soybean (*P <* 0.006) in the NGS. However, in the fertile fields, legumes fixed on average 11 and 31 kg ha^−1^ more N_2_ than in the poorly fertile fields in the SGS and the NGS, respectively (Table 3). N_2_-fixed differed significantly between sites but this varied among the legume species. N_2_-fixed by cowpea and groundnut averaged across fertility and cropping systems was 13 and 9 kg ha^−1^, respectively larger in the NGS than in the SGS while 31 kg ha^−1^ more N_2_ was fixed by soybean in the SGS.

### 3.5 N uptake and soil N balance

N uptake by sole maize was remarkably consistent for each field type in the different experimental combinations (Table 5). The combined N uptake by maize and legume in intercropping systems was larger (*P* < 0.001) than that by sole crops of maize and legumes (Table 5). An exception was sole soybean that had larger N uptake in the SGS but similar N uptake as the intercrop in the NGS. In general, total N uptakes (kg ha^−1^) by sole crops of cowpea (mean of 67 in SGS, 73 in NGS) and soybean (mean of 155 in SGS, 107 in NGS) were significantly larger than that of sole maize (63 in SGS, 27 in NGS). N uptake of groundnut (34 in SGS, 22 in NGS) was smaller than that of sole maize due to the poor yields of groundnut. Soybean grain N uptake was larger (*P <* 0.001) than that of cowpea, maize and groundnut, while stover N uptake was significantly larger in cowpea than in the other crops. Cowpea, soybean and maize in fertile fields had a significantly increased N uptake, with a mean of 32, 30 and 11 kg ha^−1^ more total N uptake, respectively in the SGS and 60, 89 and 15 kg ha^−1^, respectively in the NGS.

**Table 5:** Estimated grain and stover N uptakes and N harvest index (NHI) of cowpea (CP), soybean (SB), groundnut (GN) and maize (MZ) under different soil fertility status and cropping systems at sites in southern Guinea savanna (SGS) and northern Guinea savanna (NGS) of northern Ghana. N uptakes of intercrops represents the combined uptake by the legume and maize intercrop components while the intercrop NHI is for the legume component only.

Fertility	Cropping	SGS				NGS			
Status	system	Grain N	Stover N	Total N	NHI	Grain N	Stover N	Total N	NHI
		(kg ha^−1^)	(kg ha^−1^)	(kg ha^−1^)	(%)	(kg ha^−1^)	(kg ha^−1^)	(kg ha^−1^)	(%)
Fertile	Intercrop CP + MZ	66	53	119	48	62	51	113	52
	Sole CP	42	44	86	49	55	53	108	51
	Sole MZ	47	24	71	66	21	11	32	66
Poorly	Intercrop CP + MZ	44	37	81	45	24	28	52	38
fertile	Sole CP	20	28	48	42	17	21	38	45
	Sole MZ	35	20	55	64	10	7	17	59
	*SED*^a^*(system)*	3***	2***	5***		3***	2***	4***	
	*SED*^b^*(fertility)*	2***	1***	3***		4***	3***	7***	

Fertile	Intercrop SB + MZ	114	38	152	80	125	29	154	86
	Sole SB	135	31	166	81	134	23	157	85
	Sole MZ	49	25	74	66	29	13	42	69
Poorly	Intercrop SB + MZ	83	28	111	83	46	13	59	83
fertile	Sole SB	115	29	144	80	47	10	57	82
	Sole MZ	37	18	55	67	11	7	18	61
	*SED*^a^*(system)*	7***	2***	9***		9***	1***	10***	
	*SED*^b^*(fertility)*	5**	2*	7**		6***	1***	6***	

Fertile	Intercrop GN + MZ	46	30	76	47	29	16	45	60
	Sole GN	16	19	35	46	14	9	23	61
	Sole MZ	45	23	68	66	23	11	34	68
Poorly	Intercrop GN + MZ	41	28	69	47	21	14	35	61
fertile	Sole GN	16	16	32	50	11	9	20	55
	Sole MZ	35	20	55	64	10	8	18	56
	*SED*^a^*(system)*	3***	2**	5***		2***	1***	3***	
	*SED*^b^*(fertility)*	n.s.	n.s.	n.s.		1***	0.4**	1***	

*Prob. F. for site comparisons (MZ-CP)*: Grain N (*P* <** 0.001), Stover N (*P* = 0.008), Total N (*P* <** 0.001)

*Prob. F. for site comparisons (MZ-SB)*: Grain N (*P* <** 0.001), Stover N (*P* <** 0.001), Total N (*P* <** 0.001)

*Prob. F. for site comparisons (MZ-GN)*: Grain N (*P* <** 0.001), Stover N (*P* <** 0.001), Total N (*P* <** 0.001)

a Combined SED for cropping system across soil fertility.

b Combined SED for soil fertility.

* Significant at *P* <** 0.05.

** Significant at *P* <** 0.01.

*** Significant at *P* <** 0.001.

Sole maize had a significantly better soil N balance than intercrops and sole legumes at both sites (Fig. 1). Thus there was no evidence of an N sparing effect from intercropping or sole cropping of legumes. Soil N balance was comparable between intercrops and sole crops. Only the sole crop of groundnut had a significantly larger soil N balance than the intercrops in the NGS when both grain and stover were exported. Intercrops in the SGS had a mean soil N balance of −2 kg N ha^−1^, while sole legumes contributed 2 kg N ha^−1^ when only grain was exported (Fig. 1a and b). In the NGS however, the soil N balance of intercrop systems (+12 kg ha^−1^) was slightly larger than that of sole legumes (+9 kg ha^−1^) (Fig. 1c and d). Intercrops and sole legumes consistently provided negative N returns to the soil when both grain and stover were exported, except for groundnut in the NGS. A negative soil N balance of sole maize, with removal of both grain and stover, was observed only in the SGS which had significantly greater maize grain and stover yields with corresponding greater N uptakes (Table 4, Table 5). Legume residues had a relatively lower C:N ratio (cowpea: 23:1, groundnut: 29:1, soybean: 38:1) compared with the maize (63:1 in SGS, 73:1 in NGS) which will aid N mineralisation. Residues of cowpea and groundnut are likely to be mineralised faster and release N than that of soybean due to the relatively lower C:N ratio than soybean.

Crops in fertile fields had consistently significantly smaller soil N balance (Fig. 1). Legume species performed differently across sites in their contribution of net N to the soil. In the SGS, soybean contributed on average +9 kg ha^−1^ net N to the soil, +2 kg N ha^−1^ by groundnut and −11 kg N ha^−1^ by cowpea when only grain was exported. Groundnut gave a +22 kg ha^−1^ net N, +8 kg ha^−1^ by cowpea and +2 kg ha^−1^ by soybean in the NGS. However, when both grain and stover were exported, only the site in the NGS recorded a +10 kg ha^−1^ net N contributed to the soil N pool by groundnut.

## 4 Discussion

### 4.1 Soil fertility, *δ*^15^N of weed reference species and ^15^N natural abundance method

The *δ*^15^N signatures of the reference weeds varied among species, soil fertility status and site (Table 2). The reference plant is used to represent the *δ*^15^N of the soil N available to the legume test crops (Unkovich et al., 2008) – i.e. if the *δ*^15^N of the available soil N is uniform with depth and time, all reference plants should give the same value. Therefore, the different *δ*^15^N signatures of the weed species may reflect different isotopic discrimination among the species or extraction from different rooting depths. The variation could also be due to changes in the *δ*^15^N of the plant-available soil N pool in the course of the growing season and the relative differences in N uptake by the different reference weed species resulting from temporal differences in the volumes of soil explored by their roots (Cadisch et al., 2000; Chalk et al., 2016). By contrast, the differences in *δ*^15^N signatures between fertility status and sites presumably relate to different histories of fertiliser and crop residues use (the latter resulting in differences in turnover of N) or different isotopic discrimination during soil formation. Differences in N losses between fertility and sites, particularly through leaching due to the differences in clay content between fertile and poorly fertile fields, and sand content between both sites (Table 1) could contribute to the observed heterogeneity in *δ*^15^N enrichment of the reference weeds between fertility and sites. It is notable that the fertile soils at both sites and the soils in the SGS site which had greater soil organic carbon and nitrogen contents (Table 1) had consistently higher *δ*^15^N signatures.

The variability in *δ*^15^N signatures of the reference weeds (and of the different legumes, particularly in the SGS) observed within a field (Table 2) could be associated with spatial heterogeneity resulting from non-uniform application of mineral N fertilisers by farmers and uneven deposition of manure and urine by livestock which graze freely in the fields (Peoples et al., 2002; Unkovich et al., 2008). The variation could also be the outcome of differences in soil water content (Unkovich et al., 2008) and associated differences in N losses (particularly leaching and denitrification) across a field due to the mostly undulating topography of the fields created by ploughing by farmers without harrowing to level the fields.

The values observed in this study are within the range of 2.1–5.2‰ reported for reference weed species sampled from 63 farms in the Guinea savanna of northern Ghana (Naab et al., 2009). The variability in *δ*^15^N enrichment of the same reference species within a field suggests a within field variability in plant available soil N status, possibly due to non-uniform application of N fertilisers (Peoples et al., 2002). The variability and lack of consistency in *δ*^15^N enrichment within reference species is problematic for the accurate estimation of %Ndfa in farmers’ fields with the natural abundance method. However, using the mean *δ*^15^N enrichment of several reference weed species in each location is likely to give a more reliable estimate of the *δ*^15^N enrichment by the legumes and hence of N_2_-fixation (cf. Belane and Dakora, 2010).

### 4.2 Cropping system, soil fertility and shoot dry matter yield and N_2_-fixation

Legume shoot *δ*^15^N enrichment and %Ndfa were generally comparable between legumes in intercrops and in sole crops, as also observed by Ofori et al. (1987) and Van Kessel and Roskoski (1988) for cowpea. Shoot *δ*^15^N values observed in this study are close to the range of −1.5 to +1.5 in 30 field-grown cowpea genotypes measured using ^15^N natural abundance in the Guinea savanna of northern Ghana (Table 3; Belane and Dakora, 2010). The variability in legume shoot *δ*^15^N enrichment and %Ndfa values reflects the influence of environmental conditions (e.g. soil fertility and soil type) (Table 1), and suggests that poor soil fertility leads to a smaller shoot *δ*^15^N and a greater %Ndfa (Giller, 2001).

Sole legumes consistently fixed more N_2_ than intercropped legumes (Table 3). This was a result of the larger shoot dry matter yields and the corresponding greater shoot N accumulated by sole crops (Table 3), as the amount of N_2_-fixed greatly depends on shoot dry matter yield (Giller, 2001) and the accumulated shoot N (Peoples et al., 2009). Also, Konlan et al. (2015) reported a greater N_2_-fixation in sole groundnut than in groundnut intercropped with maize in the Guinea savanna of northern Ghana. Yet when the shoot dry matter yields were poor, such as in the SGS where cowpea yields were relatively smaller and groundnut which had poorer yields at both sites, the amount of N_2_-fixed was similar between sole crops and intercrops. Good soil fertility enhanced the production of shoot dry matter (Table 3), which also led to more N_2_ fixed. Our results corroborate other studies in the Guinea savanna (e.g. Yusuf et al., 2014), Western Kenya (e.g. Ojiem et al., 2007) and elsewhere (e.g. Giller and Cadisch, 1995) reporting that although low soil fertility enhances the %Ndfa, legumes on more fertile fields fix larger amounts of N_2_. The late sowing of groundnut due to the late onset of rainfall resulted in a poor shoot dry matter yield, low accumulated shoot N and a relatively small amount of N_2_ fixed, in comparison with results from other studies (cf. Dakora et al., 1987, Yusuf et al., 2014). This indicates that early sowing of groundnut is essential in this environment for good yield and N_2_ fixation. The N_2_-fixed by sole cowpea was in line with that observed in farmers’ field in the Guinea savanna of northern Ghana (Naab et al., 2009) and Nigeria (e.g. Sanginga et al., 2000, Yusuf et al., 2008). For soybean, comparable amounts of fixed N_2_ were reported by Sanginga et al. (1997) and Ogoke et al. (2003).

### 4.3 Crop yields, N uptake and net N contribution to soil fertility improvement

The more favourable soil fertility characteristics and rainfall in the SGS favoured a greater production of grain and stover of maize, soybean and groundnut but cowpea gave larger grain yields in the NGS with poor rainfall and soil fertility (Table 1, Table 4). Intercropping resulted in greater combined grain N removal (Table 5) as also observed by Hauggard-Nielsen et al. (2008). The soil N balance calculations suggest that sole maize (with a modest rate of applied N) has a positive N balance relative to the systems with legumes (Fig. 1). At first glance this is difficult to explain: legumes fix N_2_ from the atmosphere and are expected to contribute more N to the cropping systems than cereals. Yet a number of factors come into play that need consideration. The N balance as calculated is a partial balance representing the difference only between the N removed in products of grain (and stover where included) and the N added through fertiliser or N_2_-fixation. As such other inputs such as aerial deposition and losses of N through leaching, volatilization of ammonia or denitrification are not accounted for.

The N fertiliser was applied to the maize crop in equal split doses at three and six weeks after sowing when the maize was growing actively to ensure efficient uptake. Nevertheless, N recovery efficiencies from fertiliser rarely reach 50% (Ladha et al., 2005, Chikowo et al., 2010). We cannot rule out the possibility that perhaps, the N applied as urea was lost through leaching due to the sandy nature of the soils, particularly in the NGS (Table 1). Poss and Saragoni (1992) found that more than 30% of the urea applied to maize grown in Togo was lost through leaching and that accounted for more than 29% of the N outputs. Full N balance calculations for Ghana by Stoorvogel and Smaling (1990) indicated that about 30% of the N outputs were losses through leaching and gases. Elsewhere, Karlen et al. (1996) suggested that 46% of N applied in split doses to maize was lost through leaching, volatilization or denitrification. Though the urea was applied in furrows at 3 cm depth below the soil surface and covered after application we cannot also rule out possible losses through ammonia volatilisation. Cai et al. (2002) estimated up to 12% loss of urea-N applied to maize through ammonia volatilisation with a similar placement method. Thus, there is an uncertainty around the fate of the actual amount of N left in the soil to benefit a succeeding crop through partial N balance calculations. This suggests that partial N balances are an unreliable indicator of the sustainability of crop production systems (Janssen, 1999, Roy et al., 2003), as suggested by Bassanino et al. (2011) in determining sustainability of agro-environments in Italy.

It is worth noting that soil N mining with the removal of stover was more severe for systems with legumes due to greater N uptake than maize (Fig. 1). This is more pronounced for cowpea than soybean and groundnut due to greater stover yield (Table 5) as the variety used produced a large biomass with little shedding of leaves at maturity. Soybean sheds most of its leaves at maturity and groundnut gave poor residue yield. To offset soil N mining, the stover has to be retained in the fields but this is rarely done with groundnut where whole plants are harvested and shelled at home. Other issues associated with retaining of residues in the fields are discussed below.

Intercropping is known to reduce soil borne diseases (Hiddink et al., 2010). By contrast, continuous cropping of sole maize due to the more positive partial soil N balance can lead to diseases and pests build-up which can be averted or suppressed by rotating it with grain legumes (Stevenson and Van Kessel, 1996). The large C:N ratio of sole maize residues (63:1 in SGS, 73:1 in NGS) can lead to N immobilization, decreasing the N available to a succeeding cereal crop. Interactions between mixed legume-maize (low-high C:N ratio) residues resulting from intercropping may increase the rate of mineralisation of maize residues, improving the amount of mineralised N relative to sole maize to benefit subsequent crop, while improving soil microbial biomass and activity (Frimpong et al., 2011, Partey et al., 2014). The relatively smaller C:N ratio of sole legume residues (cowpea: 23:1, groundnut: 29:1, soybean: 38:1) can result in a relatively rapid N mineralization releasing N for the subsequent cereal crop (Palm et al., 2001).

The generally higher N concentration of legume residues than that of maize (Palm et al., 2001) suggests that the systems with legumes may produce better quality residues as feed for livestock and a possible better manure quality. These non-N benefits of the systems with legumes could make them more appealing to farmers than continuous sole cropping of maize, despite the more positive partial soil N balance. Nevertheless, the rapid mineralization of sole legume residues, particularly cowpea and groundnut might increase the risk of N leaching losses compared with that of sole maize or mixed legume-maize residues from intercropping. On-field grazing by free-roaming animals during the off-season could lead to removal of large amounts of the residues retained in the fields, reducing potential benefits of retaining residues. It may be worthwhile to export the residues to feed livestock and the manure applied to the fields in the subsequent season to directly benefit the succeeding crop (Franke et al., 2008). This seems an attractive option to reduce those losses by conserving the residues and associated benefits (Franke et al., 2008). Efficient handling, storage and transport of manure would be essential in this case to avoid possible nutrient losses and reduced benefits (Rufino et al., 2006).

**Fig. 2:**
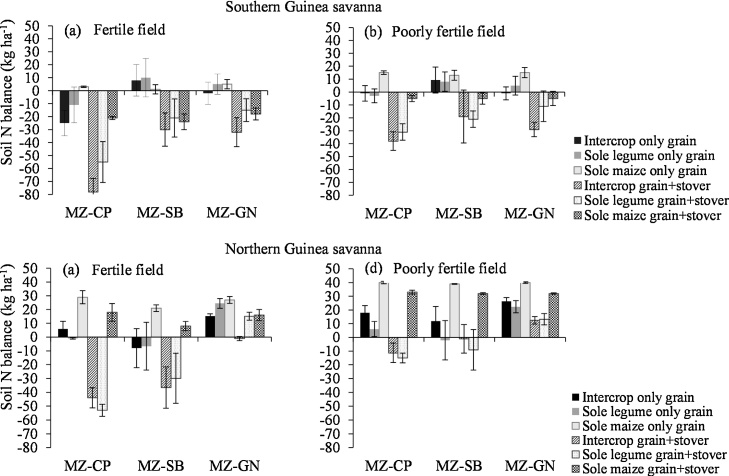
Soil N balance as influenced by different cropping systems in (a) a fertile field in SGS, (b) a poorly fertile field in SGS, (c) a fertile field in NGS and (d) a poorly fertile field in NGS of northern Ghana with grain only or both grain and stover exported. The soil N balance of intercrops combines both maize and legumes. The error bars represent the standard errors of means.

The legumes gave a different net N benefits in both agro-ecological zones, which reflected the relative %Ndfa or dependence on soil N for growth and the harvest index (HI) of the different legumes at each agro-ecological zone (Table 4). With exception of soybean, each legume species contributed a positive net N to the soil in each cropping system where the N harvest index (NHI) was smaller than the corresponding %Ndfa (Fig. 2; Table 4; data for grain N of intercropped legume only not shown). For instance, the positive net N returns to the soil by groundnut in both the SGS and NGS were due to its high %Ndfa (Table 3) and relatively low HI (compared with cowpea and soybean) which led to smaller grain N removal and NHI being smaller than the %Ndfa (Table 3, Table 5). However, groundnut gave less benefits for food and fodder than soybean and cowpea (Table 4) due to the late sowing. Therefore, in seasons with delayed onset of rainfall, it may be useful to grow relatively early maturing groundnut varieties (e.g. Edorkpo-Munikpa, 90 maturity days) in the Guinea savanna environment.

In the SGS, soybean had a higher HI than cowpea and groundnut (Table 4). Soybean also had higher %Ndfa compared with cowpea and groundnut in the SGS and soybean grown in the NGS (Table 3). However, %Ndfa of soybean was smaller than its NHI and will require 6% (intercrop) and 18% (sole crop) more Ndfa to return a net positive N to the soil. Nevertheless, with a relatively higher %Ndfa of soybean in the SGS than the NGS, combined with a high biomass production resulting in the total amount of N_2_-fixed being greater than its NHI, it contributed N to the soil in the SGS. This indicates that a positive net N input into the soil can be expected when the total amount of N_2_-fixed (kg ha^−1^) by a grain legume is greater than its NHI even if the %Ndfa is smaller than the NHI. Cowpea relied more on soil N for growth in the SGS, had a higher HI compared with groundnut with corresponding larger grain N exported (NHI > %Ndfa and total N_2_-fixed), hence a net deficit N returns to the soil (Fig. 2). Though cowpea HI and grain N removal were comparable between both sites, a relatively larger reliance on atmospheric N_2_-fixation for growth by cowpea grown in the NGS than the SGS and its NHI being smaller than the %Ndfa (Table 3, Table 4, Table 5) led to a positive net N returns to the soil in the NGS (Fig. 2). The different performance of cowpea and soybean (N_2_-fixation, grain and stover yields) across the contrasting environments in the Guinea savanna confirms the need to target the legume species to specific environments within the Guinea savanna.

The differences in rainfall and soil fertility characteristics between the two trial sites are in line with the differences in rainfall pattern (SRID, 2016) and soil fertility features (Jayne et al., 2015) between the SGS and the NGS. This suggests that the selected sites and the results are fairly representative of each agro-ecological zone in the Guinea savanna of northern Ghana. Nevertheless, trials in multiple sites within each agro-ecological zone are needed to validate the differential performance and benefits of cowpea and soybean in the contrasting environments. The net N contributed by sole legumes in this study fall within ranges reported by previous studies in the West African Guinea savanna where only grain is exported (cf. Sanginga et al., 2000 for cowpea; Ogoke et al., 2003 for soybean; Yusuf et al., 2014 for groundnut).

The amount of N_2_-fixed was larger in fertile fields (Table 3), but greater yields and a larger amount of N exported in grain (Table 4, Table 5) led to a smaller soil N balance compared with poorly fertile fields (Fig. 1). This indicates a trade-off between grain production for food and soil fertility improvement by grain legumes as demonstrated by Ojiem et al. (2007), which also depend on the legume variety (e.g. dual-purpose or grain variety). Such competing objectives need to be considered in choosing fields and legume varieties for production in the Guinea savanna. The results show a better potential for net N benefit by growing grain legumes in poorly fertile fields (Fig. 1). Yet, greater input of residues by legumes grown in fertile fields (Table 4) may enhance soil fertility by improving soil structure, microbial biomass and quantity of mineralized N to benefit subsequent cereal crops than in poorly fertile fields. The potential benefits of growing legumes may thus be limited in poorly fertile fields as also observed by Ojiem et al. (2007).

## 5 Conclusions

Intercropping or sole cropping of grain legumes have little effect on the %Ndfa but the higher density and larger area cultivated to sole legumes lead to greater shoot dry matter and amount of N_2_-fixed in sole crops. Even though %Ndfa is enhanced by growing legumes in poorly fertile fields, the overall benefits of growing grain legumes in those fields are limited as compared with the fertile fields. The results suggest that soybean can be targeted in the SGS and cowpea in the NGS for both household food and soil fertility maintenance. Groundnut is suited to both environments but growing of early maturing varieties may be essential for improved yields and soil fertility enhancement when the start of the rainy season delays. The uncertainty that surrounds calculated partial N balances of cropping systems raises issues about the extent of their usefulness and shows that partial N balances are unrealistic indicators of the sustainability of cropping systems.
